# 2,2′-[5-Bromo-*o*-phenyl­enebis(nitrilo­methyl­idyne)]diphenol

**DOI:** 10.1107/S1600536809011581

**Published:** 2009-04-08

**Authors:** Jing Gao, Yan Cheng

**Affiliations:** a Department of Pharmacy, Mudanjiang Medical University, Mudanjiang 157011, People’s Republic of China

## Abstract

A new tetra­dentate unsymmetrical Schiff base, C_20_H_15_BrN_2_O_2_, has been synthesized from 4-bromo-*o*-phenyl­enediamine and salicylaldehyde in refluxing ethanol. The dihedral angles between the two hydroxy­phenyl rings and the bromo-*o*-phenyl­enediiminatoin group are 68.6 (1) and 8.7 (1)°; the dihedral angle between the two hydroxy­phenyl rings is 70.3 (1)°. There are two relatively strong intra­molecular of O—H⋯N hydrogen bonds.

## Related literature

For the biological activity of Schiff bases, see: Boskovic *et al.* (2003 ▶); Koizumi *et al.* (2005 ▶); Oshiob *et al.* (2005 ▶). For related structures, see: Kannappan *et al.* (2005 ▶); Zhang *et al.* (2003 ▶).
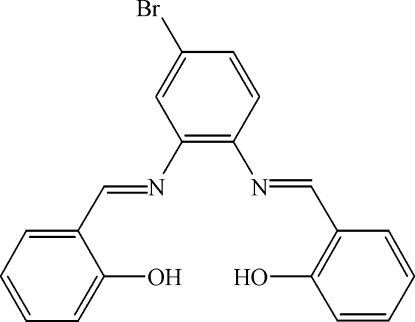


## Experimental

### 

#### Crystal data

C_20_H_15_BrN_2_O_2_
*M* *_r_* = 395.25
Monoclinic, 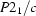

*a* = 12.8744 (10) Å
*b* = 5.9968 (10) Å
*c* = 22.106 (2) Å
β = 91.221 (1)°
*V* = 1706.3 (3) Å^3^
*Z* = 4
Mo *K*α radiation
μ = 2.43 mm^−1^
*T* = 297 K
0.12 × 0.10 × 0.08 mm

#### Data collection

Bruker APEXII CCD area-detector diffractometer
Absorption correction: multi-scan (*SADABS*; Bruker, 2001 ▶) *T*_min_ = 0.760, *T*_max_ = 0.830
8088 measured reflections
3009 independent reflections
2140 reflections with *I* > 2σ(*I*)
*R*_int_ = 0.027

#### Refinement

*R*[*F* ^2^ > 2σ(*F* ^2^)] = 0.045
*wR*(*F* ^2^) = 0.141
*S* = 1.00
3009 reflections
228 parameters
2 restraints
H-atom parameters constrained
Δρ_max_ = 0.78 e Å^−3^
Δρ_min_ = −0.62 e Å^−3^

### 

Data collection: *APEX2* (Bruker, 2004 ▶); cell refinement: *SAINT-Plus* (Bruker, 2001 ▶); data reduction: *SAINT-Plus*; program(s) used to solve structure: *SHELXS97* (Sheldrick, 2008 ▶); program(s) used to refine structure: *SHELXL97* (Sheldrick, 2008 ▶); molecular graphics: *SHELXTL* (Sheldrick, 2008 ▶); software used to prepare material for publication: *SHELXTL*.

## Supplementary Material

Crystal structure: contains datablocks I, global. DOI: 10.1107/S1600536809011581/fl2239sup1.cif

Structure factors: contains datablocks I. DOI: 10.1107/S1600536809011581/fl2239Isup2.hkl

Additional supplementary materials:  crystallographic information; 3D view; checkCIF report

## Figures and Tables

**Table 1:** Hydrogen-bond geometry (Å, °)

*D*—H⋯*A*	*D*—H	H⋯*A*	*D*⋯*A*	*D*—H⋯*A*
O2—H2*A*⋯N2	0.82	1.87	2.578 (4)	145
O1—H1⋯N3	0.82	1.90	2.614 (5)	145

**Fig. 1.**
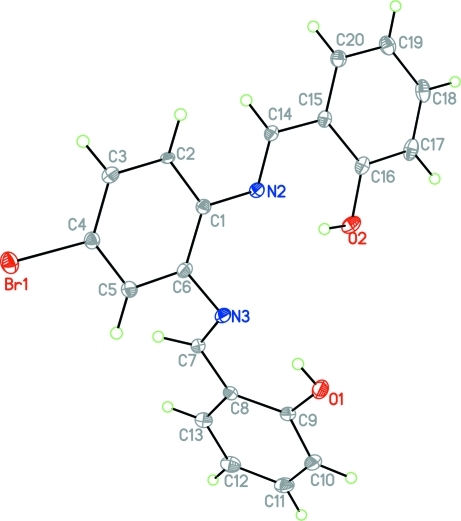
A view of the structure of (I), showing the atmoic numbering scheme and 30% probability displacement ellipsoids.

## supplementary crystallographic information

### Comment

During the past decades, Schiff bases have been intensively studied due to their strong coordination capability as well

as their diverse biological activities, such as antibacterial, antitumor, *etc*. (Koizumi *et al.*, 2005; Boskovic *et al.*, 2003; Oshiob *et al.*, 2005). The halide groups in schiff base ligands can effectively optimize the properties of the coordination complexes.

X-ray diffraction analysis indicates that (I) is a unsymmetrical Schiff (Fig. 1). The imide bond lengths of 1.276 (5) and 1.280 (5) Å for C(7)—N(3) and C(14)—N(2) are slightly longer than that found in 4-Bromo-2-(2-pyridylmethyliminomethyl)phenol (1.269 (4) Å) (Zhang *et al.*, 2003) .There are two relatively strong intramolecular hydrogen bonds (Table 1), which are similar to its derivative 4-Bromo-2-(2-pyridylmethyliminomethyl)phenol (Zhang *et al.*,2003).

### Experimental

(I) was prepared according to the method reported in the literature (Kannappan *et al.*, 2005). 4-bromo-*o*-phenylenediamine (2.16 g, 0.02 mol) was added to a stirred ethanol solution of salicylaldehyde (3.04 g, 0.02 mol (10 ml). The reaction mixture was stirred for about 3 h and then the mixture was allowed to stand at room temperature for about two days. Yellow cystals suitable for X-ray diffraction analysis were then collected with a yield of 25%.

### Refinement

H atoms bound to C and O atoms were visible in difference maps and were placed using the HFIX commands in *SHELXL97*. All H atoms were allowed for as riding atoms (C–H 0.97 Å, O–H 0.86 Å) with the constraint *U*_iso_(H) = 1.5*U*_eq_(methyl carrier), 1.5*U*_eq_(O) and 1.2*U*_eq_(carrier) for all other H atoms.

### Crystal data

**Table:**

C_20_H_15_BrN_2_O_2_	*F*(000) = 800
*M**_r_* = 395.25	*D*_x_ = 1.539 Mg m^−^^3^
Monoclinic, *P*2_1_/*c*	Mo *K*α radiation, λ = 0.71073 Å
Hall symbol: -P 2ybc	Cell parameters from 3171 reflections
*a* = 12.8744 (10) Å	θ = 1.6–25.5°
*b* = 5.9968 (10) Å	µ = 2.42 mm^−^^1^
*c* = 22.106 (2) Å	*T* = 297 K
β = 91.221 (1)°	Block, yellow
*V* = 1706.3 (3) Å^3^	0.12 × 0.10 × 0.08 mm
*Z* = 4	

### Data collection

**Table:**

Bruker APEXII CCD area-detector diffractometer	3009 independent reflections
Radiation source: fine-focus sealed tube	2140 reflections with *I* > 2σ(*I*)
graphite	*R*_int_ = 0.027
φ and ω scans	θ_max_ = 25.1°, θ_min_ = 1.6°
Absorption correction: multi-scan (*SADABS*; Bruker, 2001)	*h* = −14→15
*T*_min_ = 0.760, *T*_max_ = 0.830	*k* = −6→7
8088 measured reflections	*l* = −24→26

### Refinement

**Table:**

Refinement on *F*^2^	Primary atom site location: structure-invariant direct methods
Least-squares matrix: full	Secondary atom site location: difference Fourier map
*R*[*F*^2^ > 2σ(*F*^2^)] = 0.045	Hydrogen site location: inferred from neighbouring sites
*wR*(*F*^2^) = 0.141	H-atom parameters constrained
*S* = 1.00	*w* = 1/[σ^2^(*F*_o_^2^) + (0.085*P*)^2^ + 0.9153*P*] where *P* = (*F*_o_^2^ + 2*F*_c_^2^)/3
3009 reflections	(Δ/σ)_max_ = 0.002
228 parameters	Δρ_max_ = 0.78 e Å^−^^3^
2 restraints	Δρ_min_ = −0.62 e Å^−^^3^

### Special details

**Table:**

Geometry. All e.s.d.'s (except the e.s.d. in the dihedral angle between two l.s. planes) are estimated using the full covariance matrix. The cell e.s.d.'s are taken into account individually in the estimation of e.s.d.'s in distances, angles and torsion angles; correlations between e.s.d.'s in cell parameters are only used when they are defined by crystal symmetry. An approximate (isotropic) treatment of cell e.s.d.'s is used for estimating e.s.d.'s involving l.s. planes.
Refinement. Refinement of *F*^2^ against ALL reflections. The weighted *R*-factor *wR* and goodness of fit *S* are based on *F*^2^, conventional *R*-factors *R* are based on *F*, with *F* set to zero for negative *F*^2^. The threshold expression of *F*^2^ > σ(*F*^2^) is used only for calculating *R*-factors(gt) *etc*. and is not relevant to the choice of reflections for refinement. *R*-factors based on *F*^2^ are statistically about twice as large as those based on *F*, and *R*- factors based on ALL data will be even larger.

### Fractional atomic coordinates and isotropic or equivalent isotropic displacement parameters (Å^2^)

**Table:**

	*x*	*y*	*z*	*U*_iso_*/*U*_eq_	
Br1	0.27473 (4)	0.20430 (9)	1.05914 (2)	0.0635 (2)	
C1	0.1838 (3)	0.8216 (6)	0.94539 (16)	0.0417 (9)	
C2	0.1078 (2)	0.7023 (5)	0.97413 (14)	0.0317 (8)	
H2	0.0386	0.7461	0.9718	0.038*	
C3	0.1377 (3)	0.5168 (5)	1.00639 (16)	0.0481 (10)	
H3	0.0878	0.4316	1.0255	0.058*	
C4	0.2398 (3)	0.4554 (7)	1.01079 (17)	0.0447 (9)	
C5	0.3167 (3)	0.5727 (7)	0.98216 (18)	0.0486 (10)	
H5	0.3860	0.5298	0.9853	0.058*	
C6	0.2863 (3)	0.7566 (7)	0.94862 (18)	0.0446 (10)	
C7	0.4151 (3)	0.7950 (7)	0.87700 (19)	0.0464 (10)	
H7	0.4002	0.6486	0.8660	0.056*	
C8	0.4988 (3)	0.9113 (7)	0.84710 (18)	0.0449 (9)	
C9	0.5316 (3)	1.1220 (8)	0.8668 (2)	0.0517 (10)	
C10	0.6197 (4)	1.2177 (9)	0.8394 (2)	0.0662 (13)	
H10	0.6438	1.3558	0.8528	0.079*	
C11	0.6688 (4)	1.1140 (10)	0.7950 (2)	0.0676 (13)	
H11	0.7260	1.1814	0.7776	0.081*	
C12	0.6359 (4)	0.9088 (10)	0.7746 (2)	0.0663 (13)	
H12	0.6706	0.8387	0.7434	0.080*	
C13	0.5525 (3)	0.8076 (8)	0.79998 (19)	0.0547 (11)	
H13	0.5309	0.6685	0.7860	0.066*	
C14	0.0595 (3)	1.0603 (7)	0.90197 (16)	0.0425 (9)	
H14	0.0075	0.9705	0.9174	0.051*	
C15	0.0308 (3)	1.2554 (7)	0.86720 (17)	0.0436 (9)	
C16	0.1076 (3)	1.3984 (7)	0.84457 (16)	0.0485 (10)	
C17	0.0765 (4)	1.5884 (8)	0.81293 (19)	0.0637 (13)	
H17	0.1263	1.6840	0.7975	0.076*	
C18	−0.0274 (5)	1.6360 (8)	0.8042 (2)	0.0671 (14)	
H18	−0.0468	1.7656	0.7839	0.081*	
C19	−0.1025 (4)	1.4960 (8)	0.8251 (2)	0.0629 (12)	
H19	−0.1724	1.5278	0.8180	0.076*	
C20	−0.0733 (3)	1.3075 (7)	0.85666 (19)	0.0525 (10)	
H20	−0.1243	1.2131	0.8713	0.063*	
N2	0.1546 (2)	1.0078 (6)	0.91204 (14)	0.0425 (8)	
N3	0.3615 (2)	0.8877 (6)	0.91782 (16)	0.0501 (8)	
O1	0.4841 (3)	1.2338 (6)	0.91170 (19)	0.0761 (11)	
H1	0.4403	1.1533	0.9269	0.114*	
O2	0.2093 (2)	1.3554 (6)	0.85188 (15)	0.0654 (9)	
H2A	0.2175	1.2538	0.8764	0.098*	

### Atomic displacement parameters (Å^2^)

**Table:**

	*U*^11^	*U*^22^	*U*^33^	*U*^12^	*U*^13^	*U*^23^
Br1	0.0748 (4)	0.0534 (3)	0.0618 (3)	0.0087 (2)	−0.0108 (2)	0.0073 (2)
C1	0.040 (2)	0.041 (2)	0.044 (2)	−0.0021 (17)	−0.0003 (16)	−0.0050 (18)
C2	0.0219 (16)	0.037 (2)	0.0360 (18)	−0.0026 (15)	0.0033 (14)	0.0062 (16)
C3	0.045 (2)	0.051 (3)	0.049 (2)	−0.0092 (19)	−0.0011 (18)	0.002 (2)
C4	0.049 (2)	0.040 (2)	0.045 (2)	−0.0010 (18)	−0.0053 (18)	−0.0017 (18)
C5	0.045 (2)	0.045 (3)	0.056 (2)	0.0010 (19)	0.0008 (19)	−0.007 (2)
C6	0.043 (2)	0.043 (2)	0.048 (2)	−0.0090 (18)	0.0065 (18)	−0.0074 (18)
C7	0.039 (2)	0.040 (2)	0.060 (3)	−0.0005 (18)	−0.0018 (19)	0.003 (2)
C8	0.038 (2)	0.044 (2)	0.052 (2)	0.0042 (18)	−0.0049 (17)	0.0056 (19)
C9	0.037 (2)	0.049 (3)	0.069 (3)	−0.0003 (19)	0.002 (2)	0.005 (2)
C10	0.049 (3)	0.060 (3)	0.089 (4)	−0.011 (2)	0.000 (3)	0.012 (3)
C11	0.047 (3)	0.085 (4)	0.071 (3)	−0.005 (3)	0.008 (2)	0.015 (3)
C12	0.055 (3)	0.092 (4)	0.053 (3)	0.005 (3)	0.011 (2)	0.007 (3)
C13	0.050 (2)	0.066 (3)	0.049 (2)	0.002 (2)	0.001 (2)	0.001 (2)
C14	0.046 (2)	0.040 (2)	0.042 (2)	−0.0061 (18)	0.0042 (17)	0.0001 (17)
C15	0.056 (2)	0.039 (2)	0.036 (2)	−0.0036 (18)	0.0029 (18)	−0.0023 (16)
C16	0.065 (3)	0.046 (2)	0.035 (2)	−0.011 (2)	0.0036 (19)	−0.0024 (19)
C17	0.103 (4)	0.048 (3)	0.041 (2)	−0.018 (3)	0.010 (2)	0.004 (2)
C18	0.109 (4)	0.045 (3)	0.046 (3)	0.010 (3)	−0.013 (3)	0.005 (2)
C19	0.074 (3)	0.056 (3)	0.058 (3)	0.012 (3)	−0.006 (2)	0.003 (2)
C20	0.057 (3)	0.050 (3)	0.051 (2)	0.000 (2)	0.000 (2)	0.001 (2)
N2	0.0408 (18)	0.0416 (19)	0.0453 (18)	−0.0045 (14)	0.0014 (14)	0.0006 (15)
N3	0.0393 (18)	0.046 (2)	0.065 (2)	−0.0065 (16)	0.0084 (16)	−0.0063 (18)
O1	0.063 (2)	0.052 (2)	0.114 (3)	−0.0116 (16)	0.028 (2)	−0.021 (2)
O2	0.060 (2)	0.072 (2)	0.065 (2)	−0.0218 (17)	0.0083 (16)	0.0092 (17)

### Geometric parameters (Å, °)

**Table:**

Br1—C4	1.895 (4)	C11—C12	1.375 (8)
C1—C6	1.377 (6)	C11—H11	0.9300
C1—C2	1.378 (5)	C12—C13	1.364 (6)
C1—N2	1.385 (5)	C12—H12	0.9300
C2—C3	1.372 (4)	C13—H13	0.9300
C2—H2	0.9300	C14—N2	1.279 (5)
C3—C4	1.367 (5)	C14—C15	1.443 (5)
C3—H3	0.9300	C14—H14	0.9300
C4—C5	1.379 (6)	C15—C20	1.391 (6)
C5—C6	1.381 (6)	C15—C16	1.409 (6)
C5—H5	0.9300	C16—O2	1.341 (5)
C6—N3	1.430 (5)	C16—C17	1.391 (6)
C7—N3	1.275 (5)	C17—C18	1.377 (7)
C7—C8	1.454 (6)	C17—H17	0.9300
C7—H7	0.9300	C18—C19	1.367 (7)
C8—C9	1.399 (6)	C18—H18	0.9300
C8—C13	1.408 (6)	C19—C20	1.377 (6)
C9—O1	1.354 (5)	C19—H19	0.9300
C9—C10	1.419 (6)	C20—H20	0.9300
C10—C11	1.333 (7)	O1—H1	0.8200
C10—H10	0.9300	O2—H2A	0.8200
			
C6—C1—C2	121.2 (3)	C12—C11—H11	119.6
C6—C1—N2	120.3 (3)	C13—C12—C11	120.2 (5)
C2—C1—N2	118.5 (3)	C13—C12—H12	119.9
C3—C2—C1	117.8 (3)	C11—C12—H12	119.9
C3—C2—H2	121.1	C12—C13—C8	120.7 (5)
C1—C2—H2	121.1	C12—C13—H13	119.6
C4—C3—C2	121.0 (3)	C8—C13—H13	119.6
C4—C3—H3	119.5	N2—C14—C15	121.7 (4)
C2—C3—H3	119.5	N2—C14—H14	119.2
C3—C4—C5	121.9 (4)	C15—C14—H14	119.2
C3—C4—Br1	118.1 (3)	C20—C15—C16	119.0 (4)
C5—C4—Br1	120.0 (3)	C20—C15—C14	120.4 (4)
C4—C5—C6	117.1 (4)	C16—C15—C14	120.6 (4)
C4—C5—H5	121.4	O2—C16—C17	119.2 (4)
C6—C5—H5	121.4	O2—C16—C15	122.1 (4)
C1—C6—C5	121.0 (3)	C17—C16—C15	118.7 (4)
C1—C6—N3	118.5 (4)	C18—C17—C16	120.6 (4)
C5—C6—N3	120.5 (4)	C18—C17—H17	119.7
N3—C7—C8	122.0 (4)	C16—C17—H17	119.7
N3—C7—H7	119.0	C19—C18—C17	121.1 (5)
C8—C7—H7	119.0	C19—C18—H18	119.4
C9—C8—C13	118.7 (4)	C17—C18—H18	119.4
C9—C8—C7	120.9 (4)	C18—C19—C20	119.2 (5)
C13—C8—C7	120.3 (4)	C18—C19—H19	120.4
O1—C9—C8	122.5 (4)	C20—C19—H19	120.4
O1—C9—C10	119.3 (4)	C19—C20—C15	121.4 (4)
C8—C9—C10	118.1 (4)	C19—C20—H20	119.3
C11—C10—C9	121.5 (5)	C15—C20—H20	119.3
C11—C10—H10	119.3	C14—N2—C1	122.6 (3)
C9—C10—H10	119.3	C7—N3—C6	118.6 (4)
C10—C11—C12	120.8 (5)	C9—O1—H1	109.5
C10—C11—H11	119.6	C16—O2—H2A	109.5

### Hydrogen-bond geometry (Å, °)

**Table:**

*D*—H···*A*	*D*—H	H···*A*	*D*···*A*	*D*—H···*A*
O2—H2A···N2	0.82	1.87	2.578 (4)	145
O1—H1···N3	0.82	1.90	2.614 (5)	145
